# Author Index for Volume 91

**DOI:** 10.1038/sj.bjc.6602327

**Published:** 2004-12-21

Aareleid, T 1263

Abecassis, J 258

Abel, F 1119, 1835

Ackermann, R 985

Adam, JF 544

Adami, J

Adem, C 237

Adlercreutz, H 99

Agelidou, A 482

Agirre, X 707

A'Hern, R 627, 1763

A'Hern, RP 829

Ahmed, N 129

Ahmed, T 855

Ahmedzai, SH 248

Ahn, YC 11

Aikou, T 666

Aitchison, M 1236

Akiyama, S 1139

Akslen, LA 1225

Al Murri, AM 1063

Alaiya, AA 319

Alaminos, M 1112

Alazawi, W 2063

Aldaz, CM 753

Aldrighetti, D 45

Alfaro, M 1269

Alinari, L 850

Allal, AS 1239

Allemani, C 1263

Allen, BJ 2086

Allen, J 1447

Alliot, C 599, 1220

Almofti, A 1131

Al-Shaiba, R 205

Altés, A 678

Altman, DG 4

Ambagtsheer, G 1610

Ambrosch, P 124

Ananthaswamy, HN 795

Andersen, JA 775

Andersson, U 1174

Ando, H 972

Andreu, EJ 707

Andrews, PA 1977

Andrianifahanana, M 1633

Andrulis, I 1910

Angèle, S 783

Angerson, WJ 205

Antill, Y 900

Antonaci, S 1964

Antón-Aparicio, L 1758

Antoniou, AC 1580

Anttila, A 935, 1978

Anttinen, J 277

Aoe, M 771

Aoyagi, Y 1316

Aparicio, J 1758

Apostolidis, C 2086

Arañes, MJ 1931

Arango, D 1931

Aravantinos, G 1639

Arbyn, M 935

Ardavanis, A 482

Ardern-Jones, A 783

Arens, N 564

Ariad, S 572

Armitage, P 1983

Arslan, AA 99

Ascoli, V 998

Ashley, S 1787, 2012

Ashley, SW 1384

Ashman James, NE 1001

Asker, K 141

Aste, N 1261

Astuti, D 1835

Atangan, LI 580

Atkin, WS 1525

Atzori, L 1261

Aubert, G 1205

Audouin, J 470

Auer, G 319

Auerbach, AD 1229

Augenlicht, LH 1931

Azria, D 1251

Azzariti, A 1964

Azzouzi, A-R 739

Baccarani, M 850

Bacus, SS 1190

Bafaloukos, D 1639

Bai, AHC 1335

Baiget, M 678

Bailey, D 1477

Baldi, PL 618

Baldini, E 45

Baldus, SE 666

Balise, RR 1910

Balkwill, F 30

Balosso, J 544

Bamford, S 355

Bander, NH 164

Banerjee, R 1066

Banham, AH 141

Bar, J 1916

Barbera, S 1996

Bargo, S 1001

Bar-Haim, E 398

Barker, G 129

Barletta, E 1996

Barnabas, R 530

Barnard, DR 1866

Barni, S 1428

Barnoski, BL 1669

Barrios, M 707

Bartels, CCM 69

Bartelt, S 1482

Bartolazzi, A 1096

Bartolini, S 1038

Bassilian, S 2094

Batra, SK 1633

Battista, G 850

Bauer, JD 447

Beadle, GF 254

Beale, P 1045

Becker, JC 1495

Beech, J 1447

Beer, TM 1425

Begleiter, A 1624

Begum, N-M 1131

Beijnen, JH 1434

Bell, JA 1532

Belluco, C 2103

Belvedere, O 1428

Bénard, J 1205

Bendahmane, B 1466

Benharroch, D 572

Benoit, E 1384

Benoy, IH 1813

Ben-Porat, L 1229

Bensing, J 1050

Benvegnù, L 2103

Beral, V 530

Beranek-Chiu, J 1482

Beretta, GD 1428

Berge, G 333, 1380

Bergenheim, AT 1174

Bergh, A 1174

Berghmans, T 2018

Berglund, G 1666

Bergman, B 688

Berkowitz, R 725

Bernd, L 1012

Berney, DM 552

Bernhard, J 1893

Berrino, F 1263

Bertelsen, K 1434

Berthou, C 1195

Berwick, M 1229

Betticher, DC 1515

Bettschart, V 459

Bettuzzi, S 1842

Bevers, RFM 607

Bieche, I 2071

Bilenker, JH 213

Bing, X 633

Binns, W 942

Birchall, M 1477

Bird, R 580

Birtle, AJ 1472

Bissett, D 50

Bjørge, T 1227, 1829

Blair, CK 1866

Blamey, RW 1532

Blann, A 1645

Blann, AD 30

Blethyn, J 23

Blomqvist, C 476

Blyweert, B 1293

Body, J-J 1703

Bøe, AS 1508

Boecker, W 434

Boeing, H 1666

Boffetta, P 1280

Bogdanovic, G 913

Boher, J-M 430

Bokemeyer, C 683, 834

Bollschweiler, E 666

Bolognesi, A 45

Bonaïti-Pellié, C 603

Bonnet, ML 1735

Bonneterre, J 1466

Bonneterre, M-E 1466

Bordron, A 1195

Boren, J 2094

Bormans, G 1947

Boros, LG 2094

Borsboom, GJJM 69

Botnen, IV 333

Bouchardy, C 720

Bouchier-Hayes, DJ 359

Bougnoux, P 1466

Boukovinas, I 482

Bourhis, J 1735

Bourne, MW 23

Boustead, GB 1853

Bowen, D 1597

Bown, SG 441, 788

Boyer, M 1045

Brabender, J 666

Bracco, L 258

Brancaccio, L 1996

Brand, RE 1633

Brandariz, A 2005

Brandl, T 504

Bratti, MC 1269

Bray, F 935

Brazil, L 77

Bredholt, G 1508

Bredholt, T 1726

Bremer, M 783

Brennan, P 1280

Brenner, B 398

Brenner, W 124

Bretzel, RG 980

Brewster, DH 459

Brindley, C 1459

Brock, C 1459

Bröcker, E-B 1495

Broeders, MJM 861

Broët, P 470

Bromwich, E 1236

Brown, DJF 1993

Brown, G 23, 580

Brown, JM 621

Brown, PJ 141

Brugières, L 603

Bründler, M-A 1239

Bruzzi, P 45

Buemi, A 1751

Buerger, H 434

Buggy, Y 1308

Buhé, V 1195

Bullock, S 783

Bunyan, DJ 1155

Burk, RD 1269

Burn, J 884

Burt, PA 639

Burton, A 4

Bush, JA 1089

Butterworth, M 1213

Butzow, R 2048

Buursma, AR 2079

Buys, S 1910

Buzzi, F 489

Bydder, S 9

Caccamo, AE 1842

Caffo, O 1996

Cai, H 1364

Caine, GJ 30

Calabrese, F 2103

Calabrò, ML 998

Calista, F 618

Callahan, R 1001

Calvet, L 1205

Cameron, F 9

Camilleri-Broët, S 470

Campagnoli, E 208

Campbell, H 1575

Campbell, R 541

Campo, L 954

Canfell, K 530

Canfield, KN 1866

Canil, CM 1005

Canini, R 850

Canna, K 541

Cannita, K 618

Canzian, F 339

Cao, HJ 178

Cao, Y 1808

Capasso, F 194

Capasso, R 194

Caporali, A 1842

Capra, S 447

Capuano, MA 1996

Carbone, DP 537

Carén, H 1119

Carmon, L 398

Carnicelli, P 489

Carnino, F 45

Carreon, J 1269

Carrington, B 1447

Cascante, M 2094

Cassidy, J 50

Castellucci, P 850

Castiglione-Gertsch, M 1893

Castillejo, JA 707

Catarino, AL 732

Catto, JWF 739

Cavallo, G 1038

Cavina, R 208

Cawkwell, L 1001

Cella, D 822

Ceribelli, A 1996

Cesta, A 1442

Cetnarskyj, R 1575

Cetto, GL 96

Chaboteaux, C 1703

Chadwick, D 855

Chakrabarti, J 954

Champigneuille, J 739

Chan, FKL 1335

Chan, MWY 1335

Chan, S 1420

Chan, Y-F 1924

Chander, SK 1399

Chandraratna, RAS 580

Chang, CJ 453

Chang, CS 453

Chang, JY 453

Chang, K-W 1551

Chao, TY 453

Chao, Y 453

Chaplain, G 1263

Chapman, L 605

Chapot, B 783

Charles, P 1228

Charvet, AM 544

Chau, I 1453

Chauvenet, L 470

Chen, J 1718, 1808

Chen, J-Y 1561

Chen, LT 453

Chen, R 651

Chen, S-L 1924

Chen, W-F 1566

Cheng, A-L 453, 1561

Cheung, M 1391

Cheung, N-KV 1112

Chi, L-Y 1551

Chiba, T 1711

Chieco-Bianchi, L 998

Chin, D 1190

Cho, Y 119

Choi, SH 11

Chompret, A 603

Chrétien, F 745

Christodoulos, K 30, 1645

Christov, C 745

Chu, SH 1335

Chung, CY 453

Chung, M-Y 1551

Church, RD 1015

Cianci, G 618

Ciepluch, H 1873

Cioffi, R 489

Clarke, DM 900

Clarke, PA 1614

Clarke, SJ 1045

Clavarino, A 254

Clavel, J 916

Clayman, B 1269

Clayton, AJ 639

Clements, J 355

Clemm, C 683

Clifford, G 935

Clohisy, DR 1858

Clynes, M 1800

Coates, AS 1893

Cobb, M 248

Cocco, P 1261

Coebergh, JW 1263

Cohen, MB 213

Colditz, GA 1604

Cole, C 627, 1651

Cole, T 884

Coleman, D 942

Coleman, N 2063

Coleman, RE1 62

Collins, J 1893

Colomer, R 2101

Colonna, M 1263

Colpaert, C 1293

Coluzzi, M 998

Comber, H 1902

Combs Jr, GF 195

Comella, P 489

Conforti, R 1038

Connolly, EM 359

Constela, M 1758

Conte, PF 45

Contiero, P 1263

Cooper, M 1459

Corde, S 544

Corner, GA 1931

Cortés-Funes, H 2005, 2101

Costea, DE 1726

Couanet, D 425

Cox, DG 339

Cox, G 1301

Cox, K 1045

Cozzolino, R 1200

Craft, AW 225

Crawford, M 621

Crichton, P 996

Crimmins, D 262

Crinò, L 1038

Crispo, A 1280

Crivellari, D 1893

Croce, CM 1571, 1669

Cropp, C 1001

Crosby, K 1190

Crosby, RM 1391

Cross, NCP 1155

Cross, P 2056

Cross, SS 739

Crotty, TB 1687

Crough, T 1880

Cuijpers, V 150

Culine, S 1466

Cull, A 884

Cunningham, D 695, 839, 1453

Curtis, CE 1155

Cussenot, O 739

Cuzick, J 2056

Czech, N 124

Dabelsteen, E 760

Dahan, M 37

Dai, DL 1089

Dai, J 164

D'Alessio, G 1200

Dallimore, NS 23

Dallol, A 2071

Daly, M 1910

Dammann, E 186

Dangers, M 186

Danson, S 639

Danzon, A 1263

Darby, S 1280

Dargan, PI 995

Date, H 771

Davey, P 651

Davey, SG 512

Davidson, R 1787

Davies, ET 817

Davies, MPA 1694

Davies, S 23

Davis, FB 178

Davis, PJ 178

Dawson, E 355

de Bono, JS 50

de Boüard, S 745

de Clive-Lowe, P 714

De Falco, S 2103

de Jong, S 150

de Jonge, MJA 1459

de Klerk, JMH 200

de Koning, HJ 69, 242, 861, 1795

de la Chapelle, A 1287

De Lorenzo, C 1200

De Moor, B 1160

De Mulder, P 1050

De Smet, F 1160

De Stavola, BL 519

de Torres, C 1112

de Vathaire, F 37

De Vleeschouwer, S 1656

de Vries, EFJ 2079

Deacon, J 942

Dearnaley, D 783

Defrance, R 2026

Deichmann, M 699

Dejardin, O 1751

del Rio, E 678

Deloulme, JC 739

Delozier, T 1466

Demaerel, P 1656

DeMarchis, L 1002

DeMelker, Y 9

Dennien, B 1045

Deplanque, G 1645

Desai, M 942

Deutsch, E 1735

Dev, IK 1391

Devereux, S 651

Di Lullo, L 1442

Di Maio, M 1996

Di Napoli, A 703, 1096

Di Stefano, F 1614

Dichgans, J 219

DiCioccio, RA 1910

Dickinson, RE 2071

Dickson, RB 1372

Dienes, HP 666

Dieras, V 1466

Digby, T 1624

Dillenburg, W 1181

Dillner, J 1227

Dimba, EAO 1726

Dimopoulos, AM 1639

Din, FVN 381

Dirix, LY 1813

Dmoszynska, A 1873

Dobashi, K 1143

Dobbs, N 30

Doerfler, W 666

Dogan, A 355

Dohoulou, N 1466

Doi, Y 1571

Dold, KM 1391

Doll, R 1, 1983

Dong, X-Y 1566

Dong, Z 1105

Donnan, P 651

Dörk, T 783

Dormer, R 1149

Dornsife, RE 1391

Dorta, FJ 1758

Dørum, A 1829

dos Santos Silva, I 519

Doughty, JC 1063

Douglas, C 225

Douglas, F 884

Dowe, A 783

Draisma, G 242

Druck, T 1669

Dubois, JB 1251

Dubois, L 1947

Duffy, MJ 1308

Duffy, SW 233

Duggan, BP 164

Duijm, LEM 1795

Dumon, J-C 1703

Duncan, PJ 1155

Dunlop, MG 381

Dunn, JR 1149

Dupont, P 1947

Durkin, ME 753

Duxbury, MS 1384

Earl, J 996

Easton, DF 783, 1580

Ebihara, Y 119

Ebrahim, S 512

Eccles, D 884, 1787

Eccles, DM 1155

Echevarria, A 297

Eden, OB 1990

Edwards, SM 783

Eeles, R 1787

Eeles, RA 783

Efird, JT 1678

Egevad, L 688

Eggermont, AMM 1610

Eiberg, H 760

Eigenbrodt, E 980

Eilers, KM 1425

Eilertsen, E 1380

Eisen, T 1763

Eisen, TG 829

Eisenbach, L 398

Ejeskär, K 1119

El Hassan, MAIA 171

Elanko, N 942

Elfving, P 327

Elie, C 470

Elleaume, H 544

Ellis, IO 1532, 1591

Elomaa, I 476

El-Rayes, BF 418

El-Rehim, DMA 1532

Elst, H 1813

Emerich, J 1916

Eng, C 1835

Eng, M 359

Engeland, A 1227

Engelen, K 1160

Engelhardt, K 1045

Engers, R 985

Engholm, G 923

Epperly, AH 1391

Eschwege, P 164

Espadinha, C 732

Esposti, RD 1038

Essink-Bot, ML 69

Estève, F 544

Euler, U 434

Evans, C 186, 942

Evans, DGR 1787

Evans, H 868

Everard, M 627

Eward, KL 262

Ewerbeck, V 1012

Faa, G 1261

Fadda, D 1261

Fairchild, CR 1669

Falcone, A 1996

Falconer, A 783

Fallowfield, L 879

Fannon, D 996

Fanti, S 850

Fares, FA 1358

Faried, A 1556

Farion, R 374

Farmer, PB 1213

Farsad, M 850

Fassina, G 2103

Favaudon, V 2026

Fayyazi, A 589

Federico, M 1263

Feliu, J 1758

Feng, H 2042

Fennelly, D 1434

Ferguson, M 1293

Ferreli, C 1261

Ferrero, J-M 613

Ferry, D 1213

Feunteun, J 603

Fey, MF 1893

Ficorella, C 618

Fiegl, M 558

Field, JK 1149, 1515

Filippelli, G 489

Finklestein, JZ 1858

Flamen, P 1656, 1947

Flanagan, A 355

Fleming, FJ 1687

Fleshman, JW 1015

Florl, AR 985

Flower, D 1970

Flynn, D 855

Fokdal, L 2034

Fonseca, E 1758

Forastiere, F 1280

Forbes, JF 1893

Forbes, S 355

Forman, D 1663

Förschler, H 219

Forsea, A-M 803

Fossan, KO 1726

Foster, C 1787

Foster, CS 1694

Fountzilas, G 1639

Fox, SB 954

Fracheboud, J 242, 861, 1795

Fraier, D 50

Frame, S 459

Franceschi, E 1038

Francini, G 601

Francis, GW 1726

Franco, CRC 297

François, E 613

Frankel, WL 1287

Fransvea, E 1964

Frasci, G 489

Frascogna, V 1735

French, DP 1887

Frias, MJ 732

Frisinghelli, M 96

Fritzer-Szekeres, M 558

Fromont, G 739

Frontini, L 1428

Fujimoto, J 466

Fujisawa, T 771

Fujishita, T 1571

Fukai, Y 1556

Fukuchi, M 1556

Fukuda, K 1543

Fukunaga, A 119

Fukunaga, K 1025

Fukuyama, Y 771

Fulford, LG 2012

Furth, PA 1372

Futagami, M 633

Futreal, PA 355

Gabbert, HE 1349

Gabriele, A 850

Gafà, L 1263

Galitiis, FD 618

Gallagher, ML 213

Gallo, C 1996

Gallo, L 45

Gammell, P 1800

Ganesan, TS 30, 1645

Ganser, A 186

Gao, F 233

Gao, S 760

Garcia, CM 1263

García, RLA 525

García-Fernández, LF 1405

García-Girón, C 1758

Garden, OJ 459

Gardner, SH 153

Garin, A 1434

Garlinghouse-Jones, K 1910

Garzotto, M 1425

Gasbarri, A 1096

Gastl, G 558

Gatta, A 2103

Gatter, KC 954

Gavin, A 1902

Gazdar, AF 771

Gaze, M 1414

Geay, JF 470

Geh, JI 844

Geilen, CC 803

Gelber, RD 1893

Genieser, H-G 186

Gentle, D 1835

Georgoulias, V 482

Gerald, WL 1112

Gerharz, CD 1349

Gerl, A 683

Gerner, C 1955

Gerritsen, WR 171

Gervaz, P 1239

Gescher, AJ 1213, 1364

Gherardi, RK 745

Giaccone, G 171

Giannelli, G 1964

Giannessi, P 45

Gibbens, I 627

Gilham, C 942

Gillatt, DA 1853

Giocanti, N 2026

Giovannini, M 96

Girre, V 470

Gislefoss, RE 1227, 1829

Gisselsson, D 327

Gitsch, G 1482

Gjertsen, BT 1726

Glimelius, B 1434

Glynn, SA 1800

Gnad, U 834

Go, VLW 2094

Gogas, H 1639

Going, JJ 714

Goldberg, JS 164

Goldhirsch, A 1893

Gómez-Pardo, M 678

Gompertz, N 30

Gonzalez, A 537

González-Barón, M 1758

González-Pérez, A 525

Goodhew, KL 305

Gopas, J 572

Gore, M 627, 1763

Gore, ME 829

Gorunova, L 327

Gossett, DR 1808

Goto, K 659

Gottesman, MM 270

Gottschlich, S 124

Goubin, A 916

Gourgou, S 1251

Govindan, R 1015

Grasl-Kraupp, B 1955

Gray, J 884, 2063

Greaves, P 1364

Greenberg, M 1858

Greenberg, NA 408

Greenman, J 1001

Greystoke, A 1651

Gridelli, C 1996

Grill, J 425

Grillner, L 913

Grillon, E 374

Grimaldi, GC 208

Groenewoud, JH 1795

Grosclaude, P 1263

Groshen, S 344

Grossi, F 1428

Gruia, G 1434

Grundy, E 907

Grünewald, K 558

Gugger, M 1515

Guglielmi, A 1428

Guida, M 489

Guillen, D 1269

Guino, E 339

Guldberg, P 760

Gullberg, B 1666

Gullick, WJ 366

Guo, P 2094

Guo, R 1808

Guo, Y 1349

Gustafsson, B 913

Hack, R 558

Hackett, S 1763

Hader, C 985

Hagiwara, A 1543

Haid, A 1782

Hakama, M 106, 935

Hales, CN 714

Hall, G 627

Hall, GD 621

Hall, J 783

Hallahan, DE 537

Halsall, I 714

Halsall, JA 765

Hamaguchi, T 1775

Hamar, P 1718

Hamdy, FC 739

Hammond, LA 580

Hampel, H 1287

Han, C 954

Han, J 1604

Han, S-Y 1669

Hancock, B 621, 695

Hankinson, SE 1604

Hansen, S 1829

Hara, I 287

Harada, M 287

Hardt, PD 980

Hardy, RJ 519

Harland, SJ 1472

Harmey, JH 359

Harries, M 627, 1651

Harrington, KJ 366, 829

Harrington, LE 1391

Harris, AL 30, 954, 1645

Harrow, A 651

Hartmann, JT 683, 834

Hartmann, O 37

Hartung, G 834

Harvey, JF 1155

Hasebe, T 1316

Hashiguchi, Y 1032

Hashimoto, H 119

Hashizume, K 972

Haugen, A 333, 1380

Haun, M 558

Haustermans, K 1947

Hawcroft, G 153

Hawkins, MM 1905

Hayakawa, N 929

Hayashi, Y 972

Hayashizaki, Y 1543

Haywood, M 942

Hazzan, D 398

Hédelin, G 1263

Hedley, DW 1166

Heenan, M 1800

Heerema, NA 1866

Hehlmann, R 834

Heighway, J 1149, 1515

Heikaus, S 1349

Heim, S 775

Heiniger, A 707

Heintze, A 589

Helenius, H 277

Hellman, K 319

Hellmann, A 1873

Hellström, A-C 319

Hellström, M 688

Hémon, D 916

Hendriks, J 1293

Henne, K 1482

Hennequin, C 2026

Henner, WD 1425

Henrich, G 504

Henriksson, R 1174

Heo, JS 11

Herbert, C 1751

Herrero, R 1269

Herrlinger, U 219

Herschbach, P 504

Hershkovich, O 111

Hettmer, S 389

Hewer, A 333

Hiai, H 1571

Hibberd, S 262

Hibi, K 1139

Hildenbrand, R 564

Hildesheim, A 1269

Hill, ADK 1308, 1687

Hill, M 1453

Hill, ME 839

Hinds, A 1575

Hinkle, KW 1391

Hirakawa, K 873

Hirayama, R 1245

Hirayama, Y 312

Hitchings, AW 552

Hitt, R 2005

Hiyama, E 972

Ho, L 2056

Hoban, P 1515

Hochhaus, A 834

Hoelscher, AH 666

Hoffmann, B 1782

Hoffmann, P 129

Hofheinz, R-D 834

Höft, S 124

Hogg, M 1447

Höglund, M 327

Holly, JMP 305

Hope, Q 783

Hopper, C 441

Hopper, JL 1910

Hopper, TM 1391

Hopwood, P 884, 1787

Horgan, P 205

Horie, H 972

Horn, O 996

Hortobagyi, GN 1190

Horwich, A 683

Hoshiyama, Y 929

Hospers, GAP 2079

Houlston, R 783

House, A 893

Hovig, E 1829

Howell, A 639

Howells, LM 1213

Hsieh, RK 453

Hsu, C 1561

Hsu, DW 1678

Hsueh, S 1924

Huang, K-C 725

Huber, B 504

Hudson, EA 1364

Huebner, K 1571, 1669

Hufnagl, K 1955

Huh, SJ 11

Hull, MA 153

Hundrup, YA 644

Hunt, J 248

Hunter, DJ 1604

Hürny, C 1893

Hus, I 1873

Hus, M 1873

Husebye, ES 1508

Hussain, SA 844

Hutchinson, PE 765

Iannelli, P 1261

Ichikawa, W 1245

Iijima, H 1143

Iizasa, T 771

Iizuka, N

Ikeda, H 673, , 1977

Ikeda, M 673, 1769, 1775

Illei, P 1112

Illiano, A 1996

Im, Y-H 18

Imai, K , 1977

Imawari, M 312

Ingle, JN 237

Innes, H 1694

Inoue, H 282, 959

Ireson, CR 1399

Isa, L 1996

Isenring, EA 447

Ishihara, Y 287

Ishikawa, T 873

Ito, H 312, 1384

Ito, K 1139

Ito, Y 673

Itoh, K 287

Itoh, T 119

Izzo, AA 194

Jack, B 605

Jacot, W 430

Jacquet, J 1045

Jäger, HR 441

Jagoutz-Herzlinger, M 1782

Jakes, RW 233

Jamerson, MH 1372

James, M 1763

James, ND 844

Jänig, U 124

Jansen, FH 1795

Jansen, SJT 56

Javanovic, Z 644

Jawniak, D 1873

Jean-Claude, BJ 1066

Jędryka, M 1916

Jefford, M 900

Jellinek, DA 262

Jellum, E 1227, 1829

Jenkins, A 627

Jenkinson, HC 1905

Jennens, R 900

Jensen, A 1488

Jepsen, P 1275

Jernberg, AG 913

Jimenez-Velasco, A 707

Jimeno, A 2005, 2101

Jin, X 1808

Jöckel, K-H 1280

Joensuu, H 2048

Joh, JW 11

Johannessen, AC 1726

Johansen, C 923

Johansson, M 1174

John, E 1910

Johnsen, SP 1275

Johnson, JH 1391

Johnson, MD 1372

Jolly, S 639

Jonassen, S 1482

Jones, DJL 1213

Jones, L 441

Jones, PH 30, 1645

Jordan, S 552

Joseph, DJ 9

Joubert, A 544

Jougla, E 916

Journe, F 1703

Judson, I 1459, 1651

Julien, C 374

Jung, CW 18

Kaempgen, E 1656

Kaetsu, T 1342

Kagami, Y 673

Kahle, B 699

Kaisary, AV 1853

Kakinuma, R 659

Kakkar, AJ 30

Kalifa, C 425

Kalofonos, C 1639

Kamidono, S 287

Kampen, W-U 124

Kamsa-ard, S 106

Kandioler-Eckersberger, D 1955

Kaneko, H 119

Kaneko, K 312

Kaneko, M 972

Kang, MK 11

Kang, WK 11, 18

Kanz, L 683

Kao, WY 453

Kariola, R 1287

Karlsson, GB 1488

Katagiri, A 312

Kato, H 1556

Kato, I 99

Kato, K 119

Katoh, H 119

Katopodis, R 1453

Kaucic, K 389

Kaufmann, H 558

Kawamata, H 1131

Kawamura, M 1342

Kawamura, N 1032

Kawarada, Y 119

Kaye, S 627, 1459, 1651

Keedwell, RG 580

Keeley, VL 248

Keenan, J 1800

Keiding, N 644

Keith, AJ 305

Kejs, AMT 923

Kelleher, DK 1181

Keller, M 504

Kelly, A 1993

Kelly, G 1687

Kelly, JD 164

Kemp, BE 129

Kenny, L 254

Kersting, C 434

Khan, FA 1853

Khanfir, K 1735

Kiely, M 893

Kiesel, L 434

Kiessling, R 688

Kikuchi, S 929

Kim, DY 11

Kim, HY 18

Kim, JH 18

Kim, K 18

Kim, LS 795

Kim, S 11

Kim, YI 11

Kin, S 1543

King, P 1477

Kinlen, L 1

Kinoshita, T 1316

Kjaer, M 1434

Klein, C 1415

Klemi, P 277

Klijn, JGM 69

Klimpfinger, M 1955

Kloczko, J 1873

Kloer, HU 980

Knappskog, PM 1508

Knight, L 504

Koch, SV 923

Kodama, T 659

Kodera, Y 1139

Koenig, KL 99

Kogner, P 1835

Kolb, H-J 186

Kolch, W 186

Kollmannsberger, C 683

Kondo, S 119

Kondo, T 929

Konerding, MA 1181

Konishi, K 312

Konopka, L 1873

Konstantiniuk, P 1782

Korsmeyer, SJ 1372

Koskela, P 1227

Kosmidis, P 1639

Kouroussis, C 482

Kramar, A 1251

Kraszewska, E 1916

Kraus, S 1181

Kreil, S 834

Krex, D 2071

Krogdahl, A 760

Krona, C 1119, 1835

Kronqvist, P 277

Kros, JM 745

Krugmann, J 558

Kruyt, FAE 171

Kubota, K 659

Kugler, F 1782

Kuh, DJ 519

Kühl, J 1656

Kui, B 1293

Kumagai, K 1342

Kumar, M 552

Kumar, R 1391

Kumar, S 30

Kumekawa, Y 312

Kunitoh, H 659

Kunz, L 788

Kuopio, T 277

Kuppens, IELM 1434

Kupryjańczyk, J 1916

Kurahashi, T 312

Kuriu, Y 1543

Kurokawa, T 119

Kurokawa, Y 1711

Kurozumi, M 498

Kurth, K-H 607

Kusano, M 312, 1342

Kushima, M 312

Kutler, D 1229

Kuwano, H 1556

Labianca, R 1428

Laccetti, P 1200

Ladisch, S 389

Lafitte, J-J 2018

Lagarde, N 1195

Lahmann, PH 1666

Lakhani, SR 2012

Lalli, A 1442

Laman, H 1753

Lamb, GWA 1236

Lamb, JH 1213

Lamy, P-J 430

Landuyt, W 1947

Lane, DP 1488

Langseth, H 1829

Lannigan, A 1063

Larre, S 739

Lassus, H 2048

Latif, F 1835, 2071

Latteri, F 208

Lau, JP 1166

Laudien, M 124

Launoy, G 1751

Laurent, G 1703

Laurier, D 916

Lawlor, DA 512

Lazovich, D 1866

Le Bas, JF 544

Le Tourneau, A 470

Lebidois, J 37

Leclercq, G 1703

Lee, JE 11

Lee, MH 18

Lee, S-H 18

Lee, SI 18

Lee, ST 2094

Lee, T-C 1551

Lee, W-NP 2094

Leen, E 205

Lefebvre, P 1434

Lehtinen, M 1227

Leite, V 732

Leith, MK 1624

Lelouch-Tubiana, A 425

Lemaire, F 258

Lemanski, C 1251

Leminen, A 2048

Lemonnier, FA 398

Lentjes, EGWM 200

Lenz, H-J 344

Leong, T 1019

Lesser, M 1931

Leung, WK 1335

Lev, DC 795

Levy, A 572

Levy, D 262

Lewanski, CR 366

Lewis, IJ 225

Li, G 1089

Li, Y 2086

Liang, X-J 270

Liang, Y 1800

Lie, AK 1829

Lieb, W 1495

Liebmann, J 1349

Liggins, AP 141

Liloglou, T 1149

Lim, CK 1213

Lim, DH 11

Lim, JD 1019

Lim, S 2094

Lim, SH 1597

Lin, H-Y 178

Lin, J 1808

Lin, K-H 1924

Lin, S-C 1551

Linardou, H 1639

Linch, D 695

Lind, MJ 1851

Ling, M-T 2042

Lips, CJM 200

Lise, M 2103

Lissner, L 1666

Litjens, M 1050

Little, G 651

Liu, C-J 760, 1551

Lo, AWI 1335

Lo, KW 1335

Loisel, S 1195

Lomas, M 1758

Londesborough, P 2056

Loneragan, R 1045

Loos, W 1459

Lopes, A 2056

López-Martín, A 2005

López-Oliva, JM 1405

LoRusso, PM 418

Losada, A 1405

Lothaire, P 2018

Lothe, RA 775

Lou, P-J 441

Low, SH 829

Lowenfels, AB 811

Lowrie, AG 1327

Lu, B 537

Ludes-Meyers, J 753

Ludicke, F 720

Lundqvist, A 688

Luostarinen, T 1227

Luoto, R 1978

Luttrell, DK 1391

Lynge, E 644

Maarouf, N 1751

MacDonald, A 1001

Macè-Lesech, J 1263

Machin, D 225

Machlenkin, A 398

Mackay, J 1019, 1575, 1787

Maclennan, K 695

Macleod, U 879

MacRobert, AJ 788

Madhusudan, S 1645

Mądry, R 1916

Magee, B 639

Maggiorella, L 1735

Magnè, ML 50

Magrini, E 1038

Maguire, TM 1308

Maher, ER 1835, 2071

Maher, J 817

Mahotka, C 1349

Main, M 50

Maione, P 1996

Maiorino, L 489

Maisonneuve, P 811

Maissi, E 1887

Maitland, NJ 1842

Major, AL 720

Makino, R 312

Maksoud, A 1880

Malkin, DM 1678

Maltzman, W 1190

Manabe, T 498

Mancarella, S 489

Manda, R 1556

Mandahl, N 327

Mandarà, M 96

Manko, J 1873

Mann, P 1364

Manno, D 998

Manson, MM 1364

Marasco, A 1096

Marchetti, P 618

Marchi, E 850

Marcuello, E 678

Mariadason, JM 1931

Marino, M 2103

Markowska, J 1916

Marmey, B 470

Marques, AR 732

Marsden, HB 1905

Marteau, TM 1887

Marten-Mittag, B 504

Martin, B 2018

Martin, J 552

Martinka, M 1089

Martinsson, T 1119, 1835

Maruyama, R 771

Marzullo, A 1096

Mascaux, C 2018

Mason, B 254

Massidda, B 489

Massion, PP 537

Massoner, A 558

Mastenbroek, DCJ 171

Masucci, G 688

Masuda, N 1556

Matsueda, S 287

Matsui, K 659

Matsumoto, H 1025

Matsumoto, N 1081

Matsumoto, T 659

Matsumura, Y 1775

Matsunaga, T 972

Matsuoka, K 287

Matsuyama, H 1025

Mattes, MJ 1500

Matthews, JP 900

Maughan, TS 23

Maune, S 124

Mayer, D 907

Mayer, F 1466

Mazurek, S 980

Mazzanti, P 1996

McArdle, CS 205, 1063

McArdle, PA 541, 1755

McCarter, R 389

McCorkell, KA 1669

McCormack, VA 519

McDade, A 1045

McDermott, E 1308

McDermott, EW 1687

McFadyen, MCE 966

McGreal, G 1308

McInerney, P 1853

McKendrick, J 1019

McKenna, E 651

McKeown, DJ 1993

McLachlan, S-A 900

McLeod, HL 1015

McMillan, A 695

McMillan, DC 205, 541, 1063, 1236, 1755, 1993

McNamee, JP 1066

McNicol, A-M 541

McPherson, K 884

Meert, A-P 2018

Meisner, C 683

Meitz, J 783

Mejirovsky, E 572

Melgard, P 2034

Mellemkjær, L 1275

Melnikova, V 795

Melvin, WT 966

Memon, AA 2034

Ménégoz, F 1751

Menoyo, A 678

Merlet, P 37

Merletti, F 1280

Merlin, J-L 1205

Meschter, O 589

Metcalf, KS 621

Metzger, R 666

Micciolo, R 96

Michael, M 1019

Michalaki, V 1228

Michel, RB 1500

Middleton, G 1453

Middleton, SJ 714

Mikami, T 1081

Milano, G 613

Mileshkin, L 900

Millán, JM 2005

Miller, AM 688

Miller, CG 1391

Millon, R 258

Mimori, K 282, 959

Mineo, J-F 1195

Miosge, N 589

Mirimanoff, RO 1251

Mischak, H 186

Mistry, P 1459

Mitamura, K 312

Mitby, PA 1858

Mitsufuji, S 1543

Miyagawa, K 1543

Miyama, M 1032

Miyamoto, M 119

Miyazaki, T 1556

Miyazono, F 666

Mizoi, T 1711

Mizoue, T 929

Mizunuma, H 633

Moiseyenko, V 1434

Mok, SC 725

Molino, A 96

Møller, H 868

Møller, S 644

Mollerup, S 333, 1380

Monaghan, J 2056

Monetti, N 850

Moniaux, N 1633

Montigon, O 374

Mönting, JS 1482

Mor, O 398

Mora, J 1112

Morales, J 1269

Morcos, M 470

Moreau, Y 1160

Morelli, MF 618

Morenghi, E 208

Moreno, V 339

Mori, M 282, 959, 1143

Morimoto, Y , 1977

Morishita, Y 1222

Morizane, C 673, 1769

Morizet, J 1205

Mork, J 1227

Morley, S 893

Morohara, K 1342

Morris, M 1835

Morris, SL 829

Morrison, PJ 1787

Mortelmans, L 1947

Morton, D 2071

Moseley, R 2063

Moser, K 699

Moss, C 627, 1763

Moss, S 413

Moullan, N 783

Mourier, A 1434

Mueller, RP 666

Muhle, C 124

Muir, K 783

Muirhead, F 50

Mulder, NH 2079

Müller, C 803

Müller, D 258, 1482

Müller, M 985

Mullin, RJ 1391

Munz, DL 193

Murakami, S 119

Murakami, Y 119

Murayama, S 282

Murchison, JT 92

Murday, V 884

Muro, K 1775

Murray, E 1893

Murray, GI 966

Musk, AW 9

Musuraca, G 850

Myers, E 1687

Myint, Y-Y 572

Nachshon-Kedmi, M 1358

Nagarajan, R 1858

Nagler, RM 111

Nagura, H 1711

Naito, K 1025

Naito, Y 1711

Najman, JM 254

Nakagawara, A 972

Nakahashi, C 1316

Nakajima, M 1556

Nakajima, T 1143

Nakao, A 1139

Nakase, Y 1543

Nakata, B 873

Nakata, S 1032

Nam, HR 11

Nanni, C 850

Nanus, DM 164

Nargund, V 552

Nascimento, AG 237

Natoli, C 618

Navarro, G 707

Navarro, M 339

Neglia, JP 1858

Negoro, S 1032

Neumann, HPH 1835

Neumann, M 1482

Newby, JC 1472

Newcombe, RG 23

Nexo, E 2034

Ng, A 1990

Ng, EKW 1335

Ng, FC 233

Ng, H-K 1105

Ng, S-K 725

Ng, S-W 725

Ngan, SYK 1019

Ni, X 725

Nicholas, C 1931

Nicholson, RI 1532

Nicol, AJ 1880

Nieda, M 1880

Nihei, Z 1245

Niho, S 659

Nikitakis, NG 1074

Nilsson, S 688

Nishida, K 959

Nishimura, S 1032

Nishino, N 1342

Nishiwaki, Y 659

Nitti, D 2103

Noguchi, M 287

Noh, JH 11

Nokihara, H 659

Nordling, S 2048

Norman, AR 783, 839, 1453

Nortier, JWR 56

Norum, J 1434

Nupponen, NN 2048

Nustad, K 1829

Nutting, CM 829

Nuzzo, A 1442

Nyberg, F 1280

Nykänen, M 277

Nyström-Lahti, M 1287

Oakley-Girvan, I 1910

Oates, J 839

Oates, JR 1453

Obel, EB 644

O'Byrne, KJ 1301

Ochiai, A 1316

O'Connell, DL 1663

O'Connor, R 1800

Oda, T 1316

O'Donnell, A 1651

O'Dwyer, PJ 213

O'Dwyer, ST 1525

Oechsle, K 834

Oettrich, K 186

Ogawa, E 498

Ogawa, K 282

Ogawa, Y 873

Ohe, Y 659

O'Higgins, N 1308

O'Higgins, NJ 1687

Ohkohchi, N 1316

Ohmatsu, H 659

Ohno, S 959

Ohnuma, N 972

Ohtani, H 1711

Ohuchi, A 1711

Ohuchi, K 1711

Ohwada, S 1222

Ojima, H 1556

Oka, M

Okai, M 1880

Okayasu, I 1081

Okazaki, Y 1543

Okeke, A 1853

Okunieff, PG 1678

Okusaka, T 673, 1769, 1775

Okushiba, S 119

Oliva, KT 129

Oliveira, MBM 297

Oliveira, R 745

Oliver, T 683

Olsen, JH 1275

Olshan, AF 1866

Olsson, H 1666

O'Neill, PA 1694

Onori, JA 1391

Opitz, A 1656

Opolon, P 1205

Orfanos, CE 803

Orlandini, C 45

Osborne, JE 765

Oshima, M 1571

Osorio, A 339

Otten, W 56

Ottesen, B 644

Ottey, M 1669

Otto, SJ 861

Øvrebö, S 333, 1380

Owens, A 62

Ozenci, V 688

Ozsahin, M 1251

Paci, E 1263

Paesmans, M 2018

Paioli, A 1038

Pai-Panandiker, A 270

Paish, CE 1532, 1591

Palamidas, P 482

Palazzolo, G 1996

Palfi, S 745

Palmborg, A 688

Palmer, DH 844

Palmerini, E 1038

Pandis, N 775

Pang, JC-S 1105

Panutsopulos, D 1149

Panza, N 489

Papadimitriou, C 1639

Papakostas, P 1639

Papotti, M 1096

Paradiso, A 1964

Pardo, FS 1678

Parish, DC 1399

Park, CH 11

Park, CK 11

Park, DJ 344

Park, J 18

Park, JO 11, 18

Park, K 18

Park, S-W 753

Park, W 11

Park, YJ 11

Park, YS 18

Parkin, DM 106

Parks, RW 459

Parra, HS 208

Parzefall, W 1955

Pastorelli, C 1261

Patmore, HS 1001

Patnick, J 530

Pavarana, M 96

Pavlenko, M 688

Payen, J-F 374

Paz, A 398

Peake, DR 844

Pedersen, AT 644

Pedersini, R 96

Peel, KR 621

Pein, F 37

Pelegrin, A 1251

Pella, N 1428

Pellizzoni, C 50

Peña, C 2005

Perey, L 1893

Perkins, AC 2086

Perks, CM 305

Perren, T 627

Perren, TJ 621

Perrone, F 1996

Persad, R 1853

Peschanski, M 745

Pession, A 1038

Pestell, KE 1614

Peters-Engl, C 1782

Peto, J 942

Petrioli, R 601

Pett, M 2063

Pettett, R 355

Pezzella, F 1293

Pharoah, PDP 1910

Pharoah, PPD 1580

Philips, Z 84

Phillips, CJ 23

Phillips, DH 333

Phillips, M 9

Piantedosi, FV 1996

Piccoli, R 1200

Pilozzi, E 703

Pinder, SE 1532, 1591

Pisa, P 688

Piver, MS 1910

Pizzada, O 627

Plets, C 1656

Plisiecka-Hałasa, J 1916

Płuźańska, A 1916

Polyzos, A 482

Ponder, BAJ 1910

Pontes, C 339

Pontisso, P 2103

Poole, C 621

Poon, WS 1105

Popescu, NC 753

Popowski, Y 720

Porcelli, L 1964

Porro, MG 50

Porteous, MEM 1575

Porzio, G 618

Potter, BVL 1399

Potter, L 765

Potting, K 1050

Poulsen, JP 1434

Prevost, AT 714

Price, JE 795

Price, KN 1893

Price, T 839

Priftakis, P 913

Pringle, JH 765

Prinsloo, I 572

Prochilo, T 45

Prosper, F 707

Puglisi, F 1428

Pujade-Lauraine, E 470

Pujol, J-L 430

Pukkala, E 1227, 1978

Pulford, K 141

Purdie, DM 1880

Purohit, A 1399

Pyle, L 1763

Qin, J 1112

Qin, S 580

Qiu, Q 1066

Qu, CF 2086

Quinn, MA 129

Quintin-Roué, I 1195

Rabouel, Y 258

Rachid, Z 1066

Radcliffe, AG 23

Radzun, HJ 589

Raevaara, TE 1287

Rager-Zisman, B 572

Raghavan, D 1003

Rais, M 1261

Raitanen, J 1978

Raja, C 2086

Rakha, EA 1591

Ramp, U 1349

Rampaul, RS 1532

Ramsahoye, B 1597

Ramus, SJ 1910

Randhawa, G 62

Rank, F 644

Rao, S 839

Rapazzotti-Onelli, M 703

Ratschiller, D 1515

Raventos-Suarez, C 1669

Raverdy, N 1263

Ravetto, PF 1990

Rea, S 1442

Reade, S 1459, 1651

Reaman, GH 1858

Recchia, F 1442

Redtenbacher, S 1782

Reed, MJ 1399

Rees, BI 23

Reeve, S 129

Rehman, F 1821

Rehman, I 739

Reibel, J 760

Reitmair, A 580

Remák, E 77, 2102

Rembacken, BJ 312

Rémy, C 374

Renehan, AG 1525

Renninger, M 219

Reynolds, C 237

Reynolds, RK 1808

Reznick, AZ 111

Ribeiro, FR 775

Rice, GE 129

Ricevuto, E 618

Richards, K 900

Richel, DJ 1434

Riebeling, C 803

Rijnsburger, AJ 69

Riley, C 129

Riley, P 844

Rimondini, S 1038

Ring, AE 2012

Rizvi, SMA 2086

Robak, T 1873

Roberts, S 254

Robertson, JFR 1532

Robinson, D 868

Robinson, DO 1155

Robison, LL 1858, 1866

Robson, L 50

Robson, M 1229

Rocco, ZCD 618

Roche-Nagle, G 359

Rodriguez, AC 1269

Rodríguez-Peralto, JL 2005

Rodríguez-Pinilla, M 2005

Roesler, M 1866

Roka, S 1782

Roman-Gomez, J 707

Romieu, G 1251

Roncalli, M 208

Ronco, G 935

Roos, A-K 688

Roque, L 732

Ross, JA 1327, 1866

Ross, PJ 839, 1453

Ross, S 879

Rosso, R 45

Rouillard, D 2026

Roychoudhuri, R 868

Rückerl, C-P 683

Ruco, L 703, 1096

Rudenstam, C-M 1893

Ruiz, M 795

Russell, ST 1742

Rustin, P 1835

Rutkowski, S 1656

Ruvoletto, MG 2103

Ryder, WDJ 639

Rzepka-Górska, I 1916

Saarto, T 476

Sabo, D 1012

Sacks, M 572

Sagara, Y 959

Saggio, G 1442

Saijo, N 659

Sainsbury, R 1754

Saito, R 1143

Sakaguchi, H 466

Sakakura, C 1543

Sakamoto, A 633

Sakamoto, T 633

Sakata, K 929

Sakiroglu, O 37

Salmo, EN 1149

Salter, DM 1327

Salvadori, B 45

Salvagni, S 1996

Samra, J 2086

Sandström, M 1174

Sant, M 1263

Santoro, A 208

Santos, A 1205

Sardari, PN 1293

Sasaki, F 972

Sasaki, Y 1245

Satagopan, JM 1229

Sato, E 466, 1711

Sato, M 1131

Satomi, S 1711

Sattar, N 1755

Sauk, JJ 1074

Saunders, MP 1447

Saussele, S 834

Savage, PB 305

Scaltriti, M 1842

Scarpino, S 703

Schaffrath-Rosario, A 1280

Schamhart, DHJ 607

Scharpé, S 1813

Schausberger, E 1955

Schedvins, K 319

Schellens, JHM 1205, 1434

Schiffman, M 1269

Schil, PV 1293

Schipper, RG 150

Schlect, D 254

Schlierbach, P 980

Schlott, T 589

Schmiegelow, K 923

Schmoll, H-J 683

Schneider, PM 666

Schoemaker, NE 1434

Schønheyder, HC 1275

Schrenk, P 1782

Schrey, MP 1821

Schulte-Hermann, R 1955

Schulz, S 1482

Schulz, TF 998

Schulz, WA 985

Schwede, F 186

Schweyer, S 589

Sciacchitano, S 1096

Scopece, L 1038

Sculier, J-P 2018

Scurr, M 1459, 1651

Seifert, H-H 985

Sekine, I 659

Selby, P 893

Selby, PJ 621

Selwood, D 1000

Selwood, DL 1977

Senff-Ribeiro, A 297

Senie, R 1910

Seydel, C 996

Seynaeve, C 69

Shaaban, AM 1694

Shabir, M 1853

Shah, R 627

Shaktour, B 537

Shalet, SM 1525

Shamsaldin, A 37

Shanmugasundaram, P 1821

Shannon, WD 1015

Sharrard, RM 1842

Shaw, D 995

Shaw, MW 1149

Shea-Simonds, J 1155

Shellito, P 839

Shemer-Avni, Y 572

Shen, D-W 270

Shen, Y-C 1561

Shendler, Y 572

Sheng, ZM 1001

Sherlaw-Johnson, C 84

Sherman, ME 1269

Shi, Q 1931

Shia, G 995

Shibata, T 1349

Shichinohe, T 119

Shiiba, K 1711

Shimada, Y 1775

Shimizu, M 1245

Shimizu, N 771

Shinkai, T 659

Shirao, K 1775

Shirota, Y 1245

Shomura, H 287

Shore, RE 99

Shurland, D-L 580

Shyr, Y 537

Siavash, H 1074

Sibaud, D 1434

Sibson, DR 1694

Sidi, D 37

Sihto, H 2048

Sillibourne, J 1155

Silva, EF 297

Simonato, L 1280

Sjöberg, R-M 1119

Sjursen, A-M 1447

Skalski, V 1166

Skarlos, D 1639

Sklar, C 1858

Skotnicki, A 1873

Sloggett, A 907

Smaaland, R 1434

Smith, A 893

Smith, AI 129

Smith, BL 1190

Smith, FO 1866

Smith, HJ 408

Smith, IE 2012

Smith, P 695, 1580

Smith, R 2086

Smith, SL 1515

Snyder, R 1893

Sobrero, A 1428

Sobrinho, LG 732

Sohda, M 1556

Sohn, TS 11

Song, E 1718

Song, YJ 2086

Sonntag, B 434

Sonoshita, M 1571

Sorensen, BS 2034

Sørensen, HT 1275

Sørensen, JA 760

Sørensen, MS 1488

Sörensen, N 1656

Soroka-Wojtaszko, M 1873

Soruri, A 589

Southgate, C 783

Sozzi, WJ 1251

Spector, LG 1866

Speed, W 50

Spinelli, R 50

Spooner, D 844

Spry, NA 9

Sriamporn, S 106

Stafford, JA 1391

Stafford, ND 1001

Stahlberg, C 644

Stanley, M 2063

Stark, D 893

Stark, LA 381

Starkhammer, H 1434

Stavrinidis, E 482

Stead, M 621

Steel, M 884

Stefoni, V 850

Stein, A 398

Steinberg, W 319

Steinhoff, C 985

Steinmassl, D 1782

Stelmachów, J 1916

Stenning, S 683

Ster, KL 1195

Stevens, MCG 1905

Stevenson, JP 213

Stevenson, LF 1488

Steward, WP 1364

Stewart, AL 639

Stewart, G 1453

Stewart, JSW 366

Stiggelbout, AM 56

Stiller, CA 1905

Stocken, DD 844

Stockler, M 1045

Stockton, DL 92, 459

Stoeber, K 262, 714

Stoehlmacher, J 344

Storstein, A 1508

Stratton, MR 355

Straume, O 1225

Strauss, S 2063

Sturm, W 558

Su, A 270

Subramanian, B 606

Sugiono, M 1853

Sugiura, T 659

Suita, S 972

Sulek, K 1873

Sung, JJY 1335

Suprun, I 572

Suschek, CV 1349

Suto, K 1245

Suzuki, M 771

Suzuki, S 1342

Swaminathan, R 106

Swanson, KR 745

Swindell, R 1447

Swinn, R 714

Swinson, DEB 1301

Syrigos, K 482, 1228

Syrigos, KN 366

Tajima, Y 1342

Tajiri, H 312

Takahashi, T 1245

Takashima, T 873

Takenaka, K 498

Taketo, MM 1571

Takezako, Y 673, 1769

Takita, J 1556

Talbot, DC 30, 1645

Tamakoshi, A 929

Tamakoshi, K 929

Tamaya, T 466

Tamura, T 659

Tanaka, F 282, 498, 959

Tanaka, Y 282

Tang, H-Y 178

Tani, M 850

Tannock, IF 1005

Tari, AM 1066

Taskinen, M-R 476

Tausch, C 1782

Taylor, C 942

Taylor, GM 1990

Teague, J 355

Tebbutt, N 839

Tebeu, P-M 720

Tedesco, A 1200

Teerlink, T 150

Teicher, BA 1977

Teixeira, MR 775

ten Bokkel Huinink, WW 1434

ten Hagen, TLM 1610

Terrazzano, G 1200

Terrier-Lacombe, M-J 1205

Terry, G 2056

Thavasu, P 30

Thelen, P 589

Theodossy, T 441

Thews, O 1181

Thliveris, JA 1624

Thomas, NS 1155

Thompson, A 651

Thomson, D 254

Thomson, J 900

Thoresen, SØ 1227

Thürlimann, B 1893

Thykjaer, T 2034

Tibben, A 69

Timmerman, D 1160

Timorek ,A 1916

Tinari, N 618

Tio, J 434

Tirmarche, M 916

Tirosh, B 398

Tisdale, MJ 408, 1742

To, KF 1335

Toepler, M 980

Tokino, T , 1977

Tokui, N 929

Tomizawa, Y 1143

Tong, JHM 1335

Toniolo, P 99

Topham, C 1453

Torevell, A 1694

Torres, A 707

Torres, L 775

Toubis, M 482

Toyoki, H 466

Toyooka, KO 771

Toyooka, S 771

Toyoshima, H 929

Toyota, M , 1977

Tretarre, B 1263

Tropé, CG 1829

Troprès, I 374

Truesdale, AT 1391

Tsao, SW 2042

Tselepatiotis, E 482

Tsuda, H 1032

Tsukada, K 1556

Tsukuda, K 771

Tubiana-Hulin, M 1466

Tulusan, AH 434

Turhan, AG 1735

Turley, H 954

Tveit, KM 1434

Twelves, C 50, 1651

Tzehoval, E 398

Uchida, D 1131

Uehara, H 119

Ueno, H 673, 1769, 1775

Uitterhoeve, RJ 1050

Umemoto, M 633

Underwood, MA 541, 1755

Ura, T 1775

Urbański, K 1916

Usel, M 720

Usinowicz, MB 1910

Uster, PS 366

Utsunomiya, T 282

Vaalburg, W 2079

Vadai, E 398

Valent, A 1205

Valente, M 2103

Valle, JW 1447

van Achterberg, T 1050

van Beek, M 1795

Van Calenbergh, F 1656

van Dam, P 1813

van de Poll-Franse, LV 242

van de Velde, CJH 56

van den Berg, J 150

Van der Auwera, I 1813

van Dijck, JAAM 861

van Dillen, IJ 2079

van Dooren, S 69

van Doorn, L 1459

van Etten, B 1610

Van Gool, SW 1656

Van Laere, S 1813

Van Marck, E 1293, 1813

van Rijn, J 150

van Schaik, P 855

van Tiel, ST 1610

van Waarde, A 2079

van Wersch, A 855

Vardy, J 1045

Vasey, PA 1236

Vassal, G 425, 1205

Vassilev, LT 1415

Vaughn, D 213

Vaupel, P 1181

Vedeler, CA 1508

Vehmanen, L 476

Veiga, SS 297

Velten, M 1751

Venturini, M 45

Verbeek, ALM 861

Verbeken, E 1947

Verburg, FA 200

Vergote, I 1160

Verheij, C 1459

Verhofstad, AAJ 150

Verkooijen, HM 720

Vermaelen, P 1947

Vermeulen, P 1293

Vermeulen, PB 1813

Vernooy, M 1050

Veronesi, A 1893

Verschoyle, RD 1213, 1364

Verschuur, AC 425

Vervoort, MM 242

Verweij, J 1459

Veslemes, M 482

Vetter, CS 1495

Vicent, JM 1758

Vile, RG 366

Villain, E 37

Vintermyr, OK 1726

Viscidi, RP 1269

Vlachonikolis, I 482

Voutilainen, R 1835

Voznyi, E 1434

Wacker, J 699

Wada, H 498

Wada, T 1025

Wadsworth, MEJ 519

Wagner, LI 822

Wallace, AM 1755, 1993

Wallace, DMA 580

Wallace, E 1575

Walton, MI 1614

Wang, C-S 1924

Wang, S 1808

Wang, SS 1269

Wang, X 2042

Wang, Y 725, 1669

Wang, Y-D 1566

Wang, Z 1597

Wardley, AM 639

Warmuth-Metz, M 1656

Warnecke-Eberz, U 666

Wasylyk, B 258

Watanabe, N 1775

Waters, JS 1453, 1763

Watson, M 884, 1787

Watson, SG 1149, 1515

Watt, GCM 879

Waugh, R 1045

Waxman, J 1228

Weatherdon, KL 1166

Webster, SJ 1970

Weiderpass, E 935

Wein, S 900

Weiss, RA 1981

Weissenberger, C 1482

Weissinger, EM 186

Welch, RS 639

Weller, M 219

Wellmann, S 1645

Wells, M 651

Wen, B 1735

Wen, K 580

Werneke, U 996

Wersäll, P 688

Wersinger, EM 1425

Weston, CL 225

Wethkamp, N 1349

Weyler, J 1293

Whang, EE 1384

Wharton, SB 262

White-Koning, ML 916

Whittemore, AS 1910

Wichmann, H-E 1280

Wiendl, H 219

Wilkins, E 1155

Wilkinson, PM 639

Willer, A 834

Williams, EMI 1263

Williams, G 254

Williams, GH 262, 714

Williams, GT 23

Williamson, KE 164

Wilson, AJ 1931

Wilson, C 1063

Wilson, G 639

Winkler, MH 1853

Winter, DL 1905

Winters, ZE 305

Witucki, G 1482

Wojcicki, JM 999

Wolff, JEA 1656

Woll, PJ 1970

Wolloscheck, T 1181

Wolska-Smolen, T 1873

Wonderling, D 884

Wong, YC 2042

Woo, LWL 1399

Wood, R 651

Woodhams, JH 788

Woods, YL 1488

Wooster, R 355

Workman, P 1614

Worm, J 760

Wrba, F 1955

Wu, MF 453

Wülfing, C 434

Wülfing, P 434

Wüst, K 699

Wyke, SM 1742

Wylie, L 92

Wynn, RF 1990

Yamada, Y 1775

Yamagishi, H 1543

Yamaguchi, S 1556

Yamamoto, N 659

Yamaoka, H 972

Yamashita, T 1245

Yamazaki, K 1342

Yamazaki, T 1342

Yan, Y 1349

Yanagihara, K 498

Yang, D 344, 1808

Yang, X-A 1566

Yannai, S 1358

Yao, A 287

Yao, H 1718

Yasui, Y 1858

Yates, PM 254

Yatsuya, H 929

Yeh, KH 453

Yeh, W-I 1551

Yokoyama, T 972

Yokoyama, Y 633

Yoneyama, K 312

Yoshida, H 1131

Yoshida, K 659

Yoshida, T 1081

Yoshihiro, S 1025

Yoshimura, T 929

Yoshinaga, K 959

Young, H 907

Young, LS 1687

Yu, J 1015, 1335

Yu, XQ 1663

Yule, R 942

Yung, L 695

Yushkov, P 1096

Zahedy, S 344

Zahlten-Hinguranage, A 1012

Zahrieh, D 1893

Zalcberg, J 900

Zalcberg, JR 1019

Zaniboni, A 1428

Zeheb, R 1678

Zeleniuch-Jacquotte, A 99

Zeltzer, L 1858

Zetter, A 366

Zhang, D 2086

Zhang, S 178

Zhang, W 344, 725

Zhang, X 2042

Zhang, Y 1597

Zhao, Y 580

Zhou, L 1105

Zhu, YM 1970

Zieliński, J 1916

Zimonjic, DB 753

Zinzani, PL 850

Ziółkowska, I 1916

Zouhair, A 1251

Zschunke, F 589

Zucali, PA 208

